# AVQS: Attack Route-Based Vulnerability Quantification Scheme for Smart Grid

**DOI:** 10.1155/2014/713012

**Published:** 2014-07-24

**Authors:** Jongbin Ko, Hyunwoo Lim, Seokjun Lee, Taeshik Shon

**Affiliations:** ^1^Information Security Technology Institute, SECUVE Inc., 801 Jnk Digital Tower, 111 Digital-ro 26gil, Guro-gu, Seoul 152-848, Republic of Korea; ^2^Department of Computer Engineering, Ajou University, 206 Worldcup-ro, Yeongtong-gu, Suwon 443-749, Republic of Korea

## Abstract

A smart grid is a large, consolidated electrical grid system that includes heterogeneous networks and systems. Based on the data, a smart grid system has a potential security threat in its network connectivity. To solve this problem, we develop and apply a novel scheme to measure the vulnerability in a smart grid domain. Vulnerability quantification can be the first step in security analysis because it can help prioritize the security problems. However, existing vulnerability quantification schemes are not suitable for smart grid because they do not consider network vulnerabilities. We propose a novel attack route-based vulnerability quantification scheme using a network vulnerability score and an end-to-end security score, depending on the specific smart grid network environment to calculate the vulnerability score for a particular attack route. To evaluate the proposed approach, we derive several attack scenarios from the advanced metering infrastructure domain. The experimental results of the proposed approach and the existing common vulnerability scoring system clearly show that we need to consider network connectivity for more optimized vulnerability quantification.

## 1. Introduction

Smart grid is spreading to our everyday life, with new services such as remote control and automated electrical demand and response. However, smart grid has a number of vulnerabilities in terms of cyber security. The security paradigms of previous electric grid were to isolate major electrical facilities from outside entry by logical and physical access restrictions. However, in a smart grid, all devices are mutually connected, and therefore they communicate with each other and also with the central control center. This provides a malicious attacker with an easy attack route to the smart grid control center. Thus, it is necessary to develop and implement a new security paradigm for considering network security.

The first consideration in this new smart grid security paradigm will be to accurately understand the security vulnerabilities and estimate the risk potential of the system. Vulnerability analysis studies have been conducted for supervisory control and data acquisition (SCADA) and power grid. However, these studies only review the vulnerability of the SCADA remote communication system [1] or conduct a vulnerability analysis for electrical engineering [2]. Vulnerability quantification is a powerful method of assessing the security reliability of a system. It objectively shows the overall safety of the system as a score rather than the abstract expressions as shown by some vulnerability analysis methods. The score helps the security administrator to monitor system vulnerability in order to maintain high security even during an attack.

However, applying a vulnerability quantification to smart grid is difficult because of the environmental differences between smart grid and legacy computer systems. A smart grid is a country-sized network composed of innumerable devices using various communication protocols. In addition, the devices have technical and functional differences from standard computers. Thus, applying a single-device vulnerability quantification scheme, such as the common vulnerability scoring system (CVSS), is not suitable for smart grid. It is necessary to develop a novel vulnerability quantification scheme that is suitable to smart grid characteristics.

In this paper, we propose a novel attack route-based vulnerability quantification scheme (AVQS) that considers the network vulnerability score (NVS) and end-to-end security. We evaluate the proposed scheme by applying attack scenarios from advanced metering infrastructure (AMI) communication use cases.

In Section 2, we will present existing vulnerability quantification schemes and discuss their unsuitability for smart grids. In Section 3, the proposed approach for quantifying security vulnerability, AVQS, will be presented. We will also provide attack scenarios from AMI communication use cases, along with experimental results and analysis in Section 4. Finally, we will conclude the paper and discuss future work in Section 5.

## 2. Existing Vulnerability Quantification Schemes

### 2.1. Common Vulnerability Scoring System

The CVSS [3] was developed by a group of corporations, including CERT/CC, Cisco, DHS/MITRE, eBay, IBM, and Microsoft, to create a standardized, open vulnerability scoring framework. CVSS offers an objective and formal procedure to the vendors and users (e.g., security administrators) for evaluating the vulnerability of the target system.

CVSS has three metric groups—base, temporal, and environmental—that consist of several metrics such as the access vector, access complexity, authentication, confidentiality impact, integrity impact, exploitability, and target distribution.

- Base group represents the intrinsic and fundamental characteristics of vulnerabilities that are constant over time and user environments.
- Temporal group represents the characteristics of vulnerabilities that change over time but not among user environments.
- Environmental group represents the characteristics of vulnerabilities that are relevant and unique to a particular user environment.

CVSS uses the base group to define and communicate the fundamental characteristics of vulnerabilities. The temporal and environmental groups are selectively used to provide contextual information that more accurately reflects the risks particular to their unique environments.

**Figure 1:**
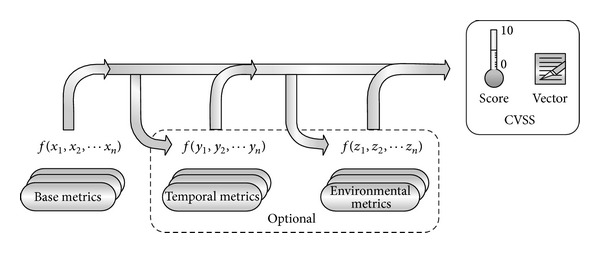
CVSS metrics and equations [3].

When the base metrics are assigned values, the base equation is used to calculate a score ranging from 0 to 10 and create a vector, as illustrated in Figure 1. The vector facilitates the “open” nature of the framework. It is a text string that contains the values assigned to each metric, and it is used to communicate exactly the derivation of the score for each vulnerability. Therefore, the vector should always be displayed with the vulnerability score [4].

### 2.2. Limitations of CVSS on Smart Grids

CVSS gives a vulnerability score for each vulnerability of a target system. This method has the ability to manage each vulnerability individually, but it is not suitable for estimating the entire system's vulnerability. Further, CVSS cannot quantify the vulnerabilities when connections occur between devices inside the system. These limitations make it impossible for CVSS to accurately quantify the security vulnerability in network systems such as smart grid, which have various types of devices that are different from a general PC infrastructure network.

### 2.3. Limitations of Existing AVQSs

There are several vulnerability quantification schemes based on attack routes or attack trees for smart grids and supervisory control and data acquisition (SCADA) [5–8]. These schemes use attack route information (number of hops, number of paths to target, etc.) as the main element for vulnerability quantification. However, the output of these schemes is expressed in varying units, for example, days, grades, degrees, and so forth. The lack of unity and public confidence in the results makes the schemes unsuitable for smart grid.

## 3. AVQS for Smart Grid

In this section, we propose a novel AVQS for smart grids. The proposed scheme first calculates the CVSS scores of the nodes on the attack route. Then, it calculates the network vulnerability score, which considers the presence of network security functions (e.g., firewalls and intrusion-detection systems (IDS)), protocol types, and communication link types. Finally, it calculates the weighted average of the total scores, considering end-to-end security functions.

### 3.1. Definitions and Assumptions

When an attack occurs on one particular target node on the network, there can be several attack routes. An attack route consists of at least two nodes: sender and receiver. In the majority of cases, a network-based attack uses the vulnerabilities and weaknesses of the intermediate nodes on the attack route. Therefore, we define two types of connections on the attack route.

- Route is the end-to-end data communication connection from a sender to a receiver. It can have several intermediate nodes.
- Section is the communication connection between two intermediate nodes on the attack route.

An attacker can use the vulnerabilities of each section to attack the route. In fact, the attacker can use local vulnerabilities for direct attacks, such as a root authority acquisition and insider attack. However, we only deal with network-based attacks, because CVSS can identify local vulnerabilities.

### 3.2. Building AVQS

**Figure 2:**
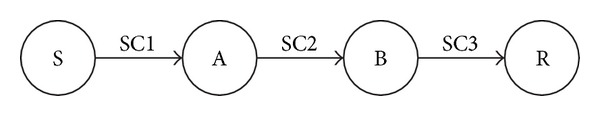
Sample attack route.

Figure 2 presents a sample attack route. In Figure 2, we assume that the attack starts from node S (sender) to the target node R (receiver). Therefore, the attack route is S, A, B, and R. In this case, there are three sections, S to A, A to B, and B to R.

As mentioned above, our scheme considers the network environments of the section. We define it as the NVS, which considers the security functions of the incoming section approaching a typical node on the attack route. In the case of Figure 2, the NVS of SC1 (Section 1) has a dependency on node A. Therefore, the AVQS score of node A is calculated by combining the CVSS of node A and the NVS of SC1:

(1) $$\begin{array}{c} \text{AVQ}{\text{S}}_{i}=\text{CVS}{\text{S}}_{i}+\text{NV}{\text{S}}_{i}\\ \left(\text{If}\;i\;\text{is}\;\text{the}\;\text{first}\;\text{node}\;\text{of}\;\text{the}\;\text{attack}\;\text{route},\text{NV}{\text{S}}_{i}=0\right). \end{array}$$

NVS consists of three types, (a) network security functions, (b) communication link types, and (c) protocol types applied on the section. Equation (2) and Table 1 show the details of NVS:

(2) NVSi=0.2×CVSSi+Si−2.4     (S=a+b+c).

The parameter constant values are defined to make vulnerability score range from 0 to 10 which is common range in CVSS. The difference between parameter constants represents the vulnerability level.

Network security functions (e.g., IDS and firewalls) have a high probability of detecting an attack through the network. Therefore, we assume that if network security functions are absent, the section has a high security-weakness score (lower is better). If one section has no network security functions, and the other sections are relatively secure (wired network and TCP/IP with security functions), it has a higher score than the rest.

The procedure for calculating NVS is as follows.

*S*_*i*_ is the sum of NVS scores (a), (b), and (c). The minimum value of *S*_*i*_ is 0.4 and the maximum value of *S*_*i*_ is 2.4. Thus, we regulate *S*_*i*_ from 0 to 2, as

(3) $$\begin{array}{c} S_{i}-0.4. \end{array}$$

As mentioned above, NVS_*i*_ has a dependency on node *i* because the security level of the section can be changed by CVSS_*i*_ itself. For example, when the calculated NVS_*i*_ has a low value (secure) and the CVSS_*i*_ has a high value (weak), it is difficult to determine the security degree. Thus, our scheme uses a floating variable to solve this problem:

(4) $$\begin{array}{c} \frac{10-\text{CVS}{\text{S}}_{i}}{5}. \end{array}$$

By applying (4), NVS_*i*_ is changed properly according to CVSS_*i*_ from −2 to 2. For example, if CVSS_*i*_ is 0, the range of NVS_*i*_ is changed from −2 to 0. Further, if CVSS_*i*_ is 10, the range of NVS_*i*_ is changed from 0 to 2.

Therefore, the final NVS_*i*_ equation is presented as follows:

(5) NVSi=Si−0.4−10−CVSSi5=0.2×CVSSi+Si−2.4(S=a+b+c).

Then, we can calculate the AVQS score for node *i* using (1). After calculating AVQS scores for every node of the attack route, we can determine the overall AVQS score of the attack route:

(6) $$\begin{array}{c} {\text{AVQS}}_{\text{avg}}=\alpha \times \frac{\sum_{i=1}^{n}{\text{AVQS}}_{i}}{n}. \end{array}$$

Here, *α* refers to end-to-end security functions, such as VPN, IPsec, and e2e encryption. These functions provide a secure communication channel from source to destination on the route. Therefore, *α* must be considered to determine an accurate vulnerability score of the attack route. However, some attackers can attack the target using specific techniques even when the function is applied. Therefore, our scheme applies the end-to-end security score to the final AVQS score. However, we make the influence of this score slight because it can be confusing. The end-to-end security scores of our scheme are shown in Table 2.

## 4. Experimental Results and Analysis

To verify the suitability of our scheme, we experimented with attack scenarios based on actual communication use cases of the AMI domain on smart grid.

### 4.1. Attack Scenarios

In order to quantify the vulnerability of the AMI group, we considered communication use cases of the AMI domain and attack scenarios that can actually occur in smart grid.

**Figure 3:**
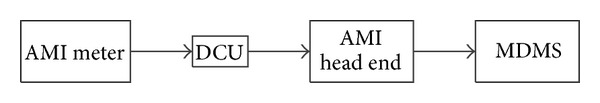
Communication flow of use case 1.

*(i) Use Case 1*. While communicating with the AMI meter, the meter data management system (MDMS) collects and stores the customers' electrical consumption information (see Figure 3).

**Figure 4:**
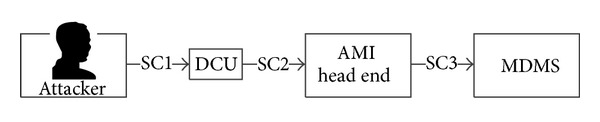
Attack route for Scenario 1.

*(ii) Attack Scenario 1*. In order to counterfeit and falsify the customers' electrical consumption information stored in the MDMS, we assume that the attacker trespasses through the data concentrator unit (DCU). In this case, the attacker can overcharge or undercharge the consumers (see Figure 4).

**Figure 5:**
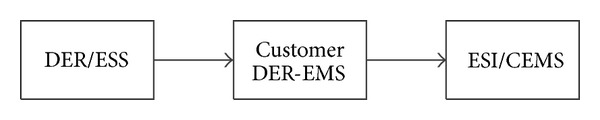
Communication flow of use case 2.

*(iii) Use Case 2*. This includes storing energy when electrical charges are low and using the energy when electrical charges are high (see Figure 5).

**Figure 6:**
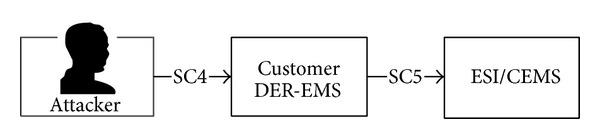
Attack route for Scenario 2.

*(iv) Attack Scenario 2*. We assume that the attacker trespasses through the customer distributed energy resources-energy management system (DER-EMS) and illegally controls the energy services interface/customer energy management system (ESI/CEMS). In this case, the attacker can cause the DER and energy storage system (ESS) to malfunction. Owing to the attack, the consumer cannot charge the DER or spend electricity stored in the ESS (see Figure 6).

### 4.2. Experimental Results

The CVSS values are calculated using NIST's “Common Vulnerability Scoring System Version 2 Calculator.” Metric values and details can be found in NIST's criteria “A Complete Guide to the Common Vulnerability Scoring System Version 2.0.” [1].

*(i) Attack Scenario 1*.  Table 3 shows the calculated CVSS values of each node of the attack route for Attack Scenario 1. For the DCU, we only use the base and temporal metrics for calculation because the environmental metric of CVSS is an optional process. 

*Access Vector*. All components consist of adjacent networks in a smart grid. 

*Access Complexity*. Specialized access conditions or situations do not exist. 

*Authentication*. Authentication is not required to access and exploit the vulnerability. 

*Confidentiality Impact*. There is considerable information outflow, but the scope of the loss is constrained. 

*Integrity Impact*. There is considerable information modification, but the scope of what the attacker can affect is limited. 

*Availability Impact*. There is no impact to the availability of the system. 

*Exploitability*. Even if the attacker is unskilled, he can easily attack vulnerabilities. 

*Remediation Level*. There is an unofficial, nonvendor solution available. 

*Report Confidence*. There is little confidence in the validity of the reports.

Because of the presence of network security functions, communication link types, and protocol types, the NVS score is assigned as shown in Table 4. For example, there are no network security functions in Section “SC1.” Further, Section “SC1” uses a wired network and a power-line communication (PLC) protocol without security.

The calculated NVS values of each section and the calculated AVQS values of each node are as follows. The NVS values are calculated by (5) and the AVQS and average AVQS values are calculated by (1) and (6), respectively. 

*(ii) Attack Scenario 2*. We calculated CVSS, NVS, and AVQS in the same manner for Attack Scenario 2, as shown in Tables 5 and 6.

The comparison of the results of AVQS and the average CVSS shown in Table 7. In the case of Attack Scenario 1, we assume that end-to-end security is applied. By (6), the average AVQS is multiplied by 0.8. However, end-to-end security is not applied in Attack Scenario 2, and therefore, we multiply by 1. As a result, the average AVQS score is 3.91 in Attack Scenario 1 and 6.06 in Attack Scenario 2.

In the case of Attack Scenario 1, the difference of the two values is 1.32 because network security functions were applied to the route. This means that our model includes the network security feature characteristics and has accurate quantification. On the other hand, we can see that the final AVQS value is bigger than the average CVSS value in the case of Attack Scenario 2, because the network security functions were minimal. This shows that our proposed scheme can provide the proper vulnerability quantification results in two cases.

## 5. Conclusion

In this paper, we proposed a novel AVQS to accurately measure the security level in a smart grid. The proposed approach includes NVS and end-to-end security functions. To verify the proposed approach, we derived attack scenarios using a few use cases from AMI communications. The evaluation results showed an obvious difference between the proposed approach and the average CVSS. The difference between the two values indicates the importance of considering network security features. Thus, we can see that the proposed scheme is a more optimized approach than the previous one. In future work, various attack scenarios on the AMI and the rest of the domains like SA (substation automation), WAMS (wide area measurement system), and DER (distributed energy resources) should be considered.

## Figures and Tables

**Table 1:** Network vulnerability scores on the section.

Type			Score
Network security functions (a)	Present		0.13
	Not present		1.20

Communication link types (b)	Wired		0.13
	Wireless		0.67

Protocol types (c)	TCP/IP	With security	0.14
		Without security	0.4
	Electricity-specific protocols	With security	0.27
		Without security	0.53

**Table 2:** End-to-end security scores.

Type		Score
End-to-end security functions	Present	0.8
	Not present	1

**Table 3:** CVSS per node of the attack route.

		DCU	AMI head end	MDMS
Base	Access vector	Adjacent network	Adjacent network	Adjacent network
	Access complexity	Low	Medium	High
	Authentication	None	Single instance	Multiple instances
	Confidentiality impact	Partial	Complete	Complete
	Integrity impact	Partial	Complete	Complete
	Availability impact	None	Partial	Complete

Temporal	Exploitability	High	Functional	Proof of concept
	Remediation level	Work around	Work around	Work around
	Report confidence	Unconfirmed	Confirmed	Confirmed

CVSS		**4.1**	**6.3**	**5.3**

**Table 4:** Network security scores and AVQS scores.

Type	SC1	SC2	SC3
Network security functions	Present	Not present	Present
Communication link type	Wired	Wired	Wired
Protocol type	Protocols for PLC without security	Protocols for PLC without security	TCP/IP with security
NVS	**−0.79**	**0.72**	**−0.95**
AVQS	**3.31**	**7.02**	**4.35**

**Table 5:** CVSS per node of the attack route.

		Customer DER/EMS	ESI/CEMS
Base	Access vector	Adjacent network	Adjacent network
	Access complexity	Medium	High
	Authentication	Single instance	Multiple instances
	Confidentiality impact	Partial	Complete
	Integrity impact	Partial	Complete
	Availability impact	Complete	Complete

Temporal	Exploitability	Functional	Proof of concept
	Remediation level	Work around	Work around
	Report confidence	Confirmed	Confirmed

CVSS		**4.1**	**5.7**

**Table 6:** Network security scores and AVQS scores.

Type	SC4	SC5
Network security functions	Not present	Not present
Communication link type	Wired	Wired
Protocol type	Protocols for PLC without security	Protocols for PLC without security
NVS	**0.60**	**0.52**
AVQS	**6.3**	**5.82**

**Table 7:** Comparison of the experimental results.

	Attack Scenario 1	Attack Scenario 2
Average CVSS	5.23	5.50
Final AVQS	3.91	6.06
